# Cellular Superspreaders: An Epidemiological Perspective on HIV Infection inside the Body

**DOI:** 10.1371/journal.ppat.1004092

**Published:** 2014-05-08

**Authors:** Kristina Talbert-Slagle, Katherine E. Atkins, Koon-Kiu Yan, Ekta Khurana, Mark Gerstein, Elizabeth H. Bradley, David Berg, Alison P. Galvani, Jeffrey P. Townsend

**Affiliations:** 1 Yale School of Public Health, New Haven, Connecticut, United States of America; 2 Global Health Leadership Institute, Yale University, New Haven, Connecticut, United States of America; 3 Department of Molecular Biophysics and Biochemistry, Yale University, New Haven, Connecticut, United States of America; 4 Yale University School of Medicine, New Haven, Connecticut, United States of America; The Fox Chase Cancer Center, United States of America

## Introduction

Worldwide, more than 250 people become infected with HIV every hour [1], yet an individual's chance of becoming infected after a single sexual exposure, the predominant mode of HIV transmission, is often lower than one in 100 [2]. When sexually transmitted HIV-1 infection does occur, it is usually initiated by a single virus, called the founder strain, despite the presence of thousands of genetically diverse viral strains in the transmitting partner [3]. Here we review evidence from molecular biology and virology suggesting that heterogeneity among CD4+ T cells could yield wide variation in the capability of individual cells to become infected and transmit HIV to other cells. Using an epidemiological framework, we suggest that such heterogeneity among CD4+ T cells in the genital mucosa could help explain the low infection-to-exposure ratio and selection of the founder strain after sexual exposure to HIV.

During sexual transmission, founder viral strains preferentially infect CD4+ T cells using the CCR5 coreceptor [4], [5]. At the time of initial exposure to HIV, these CD4+ T cells exhibit baseline heterogeneity due to stochasticity in cellular gene expression [6] and dynamic variation in immunological status (activated, resting, etc.) [7]. In addition, because CD4+ T cells are mobile, they are heterogeneously distributed in the genital mucosa, with varying degrees of clustering and contact [8]–[11]. In other contexts, it is well-known that heterogeneity among isogeneic cells inside the body can affect many cellular behaviors and outcomes, including infection dynamics [12], [13].

Epidemiological analyses of disease outbreaks among people indicate that heterogeneity in the ability of individuals in a population to spread disease can have a significant impact on whether a local outbreak becomes an epidemic [14]. Heterogeneity among a population of CD4+ T cells may play a similarly critical role in the establishment and spread of HIV in the genital mucosa after sexual exposure.

## Basic and Individual Reproductive Number

To quantify the spread of infectious disease, epidemiologists use the basic reproductive number, *R*_0_, which describes the average number of secondary infections that arise from one infected individual in an otherwise totally susceptible population [15]. The basic reproductive number can be approximated as the product of the following: (1) the average number of susceptible individuals contacted by an infected individual during the infectious period (the “number of contacts”) and (2) the average probability that a susceptible individual will become infected by a single infected individual during its infectious period (the “shedding potential”). Thus,
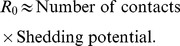



The number of secondary infections caused by a specific individual throughout the time that the individual is infectious is called the “individual reproductive number” [14]. For any disease within a given population, there exists a distribution of individual reproductive numbers, of which *R*_0_ is the mean [14]. In populations of homogeneous individuals, the distribution of individual reproductive numbers will be clustered around the population average value of *R*_0_, and thus, this average value will more accurately predict the likelihood of transmission from each infected to each susceptible individual. If *R*_0_>1, then an outbreak is likely to become an epidemic, and if *R*_0_<1, then an outbreak will not spread beyond a few initially infected individuals [15], [16].

In heterogeneous populations, however, the population average value of *R*_0_ is less predictive of transmission dynamics [14]. For example, in populations with highly right-skewed distributions of individual reproductive numbers, most individuals infect few, if any, others, but a few individuals infect many others. In such populations, there is a high probability that a disease outbreak will not be sustained in the population and will instead go extinct [14]. In some cases, however, those rare individuals in the tail of the distribution with a much higher-than-average individual reproductive number while they are infected, known as “superspreaders” [15], can have a significant impact on whether an outbreak becomes an epidemic or goes extinct. Epidemiological outbreak investigations, which track the spread of disease by a technique called contact tracing, have identified the existence of superspreaders in many well-known infectious disease outbreaks, including typhoid fever, measles, smallpox, Ebola, and severe acute respiratory syndrome (SARS) [14], [17], [18]. These rare individuals often make a significant, sometimes deciding, contribution to the dynamics of disease spread (Table 1).

**Table 1:** Superspreading events during infectious disease outbreaks.

Disease	Location (year)	(R_0_)^a^	SSE^b^	References
**EBOLA**	Congo (1995)	1.83	21+, 28–38	[85], [86]
**MEASLES**	Greenland (1951)	16	250	[15], [87]
	US (1985)	16	69,84	[15], [88]
	Canada (2011)	16	678	[15], [17]
**PNEUMONIC PLAGUE**	China (1946)	1.3	32	[89], [90]
**SARS**	Hong Kong (2003)	3	187	[91], [92]
	Vietnam (2003)	3	20	[91], [93]
	Singapore (2003)	1.6	12,21,23,23,40+	[14], [19]
	Canada (2003)	3	19,12–24	[91], [94]
**SMALLPOX**	Yugoslavia (1975)	5.5	38	[95], [96]

a R_0_: The average number of secondary cases caused by an infected individual during the outbreak; here, R_0_ is reported either for a specific outbreak, when available, or as a measure calculated based on multiple past outbreaks.

b SSE: Superspreading events—number of infections caused by a single individual during an outbreak; number of infections caused by multiple superspreading events during the same outbreak are separated by commas.

This table is adapted from the supplementary material from reference [14].

**Figure 1:**
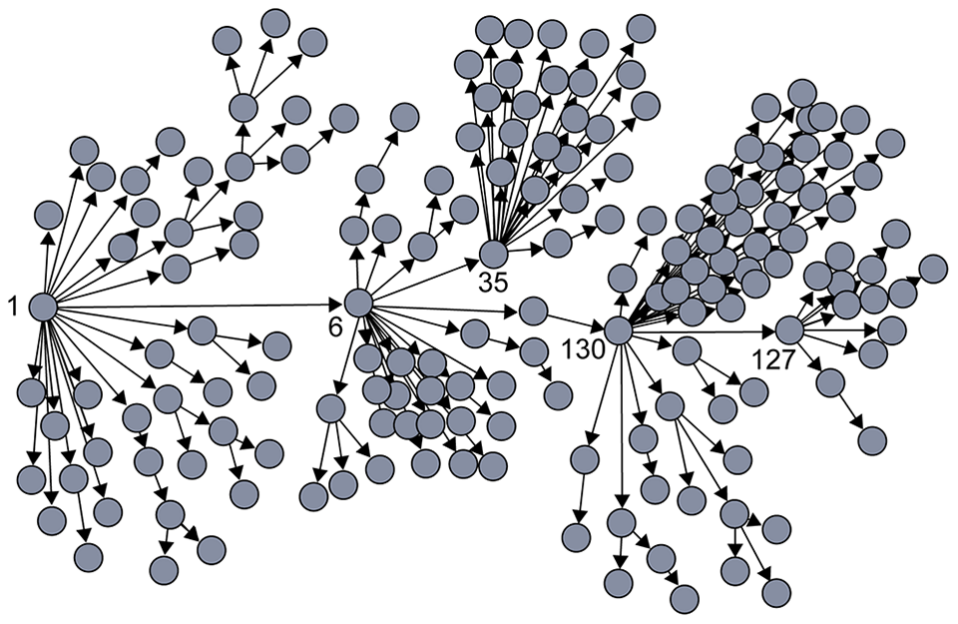
Contact tracing of SARS in Singapore showed that most people (gray circles) transmitted the virus to very few others, while a few individuals acted as “superspreaders,” infecting many more people than average. Patient numbers corresponding to those individuals who were identified as superspreaders are shown. All cases trace back to patient 1 [18].

During the 2003 SARS outbreak in Singapore, for example, the majority of individuals who became infected spread the virus either to no one else or to only one other [14]. Five infected individuals, however, were superspreaders, each infecting at least 20 others (Figure 1) [19]. In this example, *R*_0_, which is an average population value, did not adequately describe the dynamics of SARS because it did not capture the heterogeneity among individuals in their ability to spread disease or the key contribution made by superspreaders to establishment and spread of the virus [14].

## Individual Cellular Reproductive Number

For a population of HIV-infected cells inside the body, the basic reproductive number, *R*_0_ (a population average), has been quantified [20]. As with individual humans inside a population, however, empirical evidence indicates that individual human cells inside a population are also heterogeneous [12], varying in their contact with one another, their ability to become infected (permissivity), and also in whether and to what extent they transmit infectious virus to other cells during their infectious period. Such heterogeneity among CD4+ T cells in the genital mucosa of a single individual could generate a skewed distribution in the individual *cellular* reproductive number, or ICRN, in the context of HIV infection. Here we review evidence for heterogeneity among CD4+ T cells that could lead to wide variation in ICRN and possibly give rise to cellular superspreaders.

## Number of Contacts

CD4+ T cells exhibit considerable heterogeneity in activation status (e.g., resting or activated) and expression of surface molecules important for HIV infection (including the HIV coreceptors CCR5 and CXCR4) in human penile [21], foreskin [22], [23], cervical [24], [25], and rectal [26] tissue. In addition, various studies that stain for CD4+ T cells in uninfected genital mucosal tissue, such as cervical tissue [27] and human foreskin [22], indicate that T cells vary in their spatial distribution and the extent to which they form clusters. Cell density and spatial arrangement have been identified as important sources of heterogeneity among cells that can affect virus spread in vitro [8], [28]–[30]. Indeed, imaging studies exploring the dynamics of virus spread in a model of sexual transmission to female nonhuman primates indicate that virus spreads unevenly among clusters of cells in the endocervix [9]. Cells in these clusters tend to be in close proximity, and if cell-to-cell transmission is far more efficient than cell-free transmission, as some studies suggest [31], then a cell that is physically touching its neighbors could generate more secondary infections than a cell that is not close enough to others to transmit virus by direct contact [8]. Thus, heterogeneity in cell distribution and clustering inside the body could generate wide variation in the efficiency of virus transmission from cell to cell and in ICRN.

Transmission of virus from an infected to a susceptible cell also depends on a cell's permissivity to productive infection. The level of surface expression of CD4 and CCR5 (the predominant coreceptor utilized during acute infection [32]) varies widely among CD4+ T cells [25], [33], even in a single individual [34], and affects cellular permissivity to HIV [35], [36]. Indeed, low expression of CD4 or CCR5 can completely inhibit infection of CD4+ T cells by certain viral strains [37]. A recent multiparameter analysis of HIV entry efficiency at the level of single cells indicated large cell-to-cell variation in expression of CD4, CCR5, and the coreceptor CXCR4, which subsequently influenced permissivity of individual cells to HIV binding and entry [38]. In addition, CD4+ T cells isolated from rectal and cervical tissue exhibit considerable heterogeneity in expression of the surface integrin α4β7, which can specifically bind the V2 loop of the HIV envelope protein gp120 and may improve cell-to-cell spread by activating other cell-surface molecules in the viral synapse [39]. Experiments in vitro indicate that HIV preferentially infects cells expressing high levels of CCR5 and that infection can be further enhanced by high levels of surface α4β7 expression in some individuals [40], [25]. Heterogeneity among CD4+ T cells in expression of specific cell surface receptors can thus affect permissivity and cell-to-cell transfer of HIV infection in the genital mucosa.

Permissivity of cells to productive HIV infection can also be affected by intracellular proteins called restriction factors, which block the progress of HIV through the cell [41]. Expression of some cellular restriction factors has been shown to vary among different human populations [42] and even between different types of CD4+ T cells within the same individual [43]. Notably, an intracellular host restriction factor known as SAMHD1 (sterile alpha motif [SAM] and histidine/aspartic acid [HD] domain-containing protein 1) blocks reverse transcription in resting—but not activated—CD4+ T cells, inhibiting HIV replication. These findings help to explain the inability of resting CD4+ T cells to produce infectious virus [44]. HIV combats the effects of some cellular restriction factors with its own viral proteins, but the success of these proteins in overcoming cellular resistance and facilitating virus production depends on their quantity within the cell, which can also vary depending on viral gene expression levels [41].

Together, these data suggest that among CD4+ T cells in the genital mucosa, significant heterogeneity may exist in the number of contacts that become infected by any given infected cell even within the same host, potentially leading to a skewed distribution of ICRN.

## Shedding Potential

Release of infectious viral particles from an infected CD4+ T cell, or shedding potential, can be influenced by many factors in both the cell and the virus. Variation in virus gene expression at the level of individual cells has been demonstrated in vivo in a mouse model of cytomegalovirus infection [45], and heterogeneity among individual cells in the production of virus particles, or virus shedding, has been shown through analysis of cells isolated from simian immunodeficiency virus (SIV)–infected nonhuman primates, in which rare activated CD4+ T cells were shown to individually release large quantities of virus [10]. Here we focus on variability in gene expression and its potential impact on an individual cell's capability to release infectious virus as a possible source of heterogeneity in shedding potential.

Stochasticity in cellular gene expression is a common phenomenon [6] and has also been observed in viral gene expression, including HIV. In populations of genetically identical cells infected with HIV, viral genes tended to be expressed at either high or low levels but were also rarely expressed at intermediate levels, varying from cell to cell [46].

The site of virus integration also affects viral gene expression. HIV viral DNA preferentially integrates at sites of active cellular gene expression although not at any specific site or in any specific gene [47]–[49]. Some viral integration events occur at sites of much higher gene expression than others [47], and gene expression can vary significantly depending on the site of integration, even in genetically identical cells [50]. Since the majority of HIV-infected cells in lymph nodes and peripheral blood contain a single virus [51], while cells in splenic tissue contain one to eight integrated proviruses (mean 3.2) [52], viral integration site can be an important factor in determining viral gene expression and likely also subsequent virus shedding potential from any given cell.

Within the population of CD4+ T cells in the genital mucosa, therefore, a wide range of ICRN may exist due to stochastic and/or infection-driven variations in viral gene expression and viral particle production, arising from potentially wide heterogeneity in virus shedding potential among infected cells.

## Cellular Superspreaders

**Figure 2:**
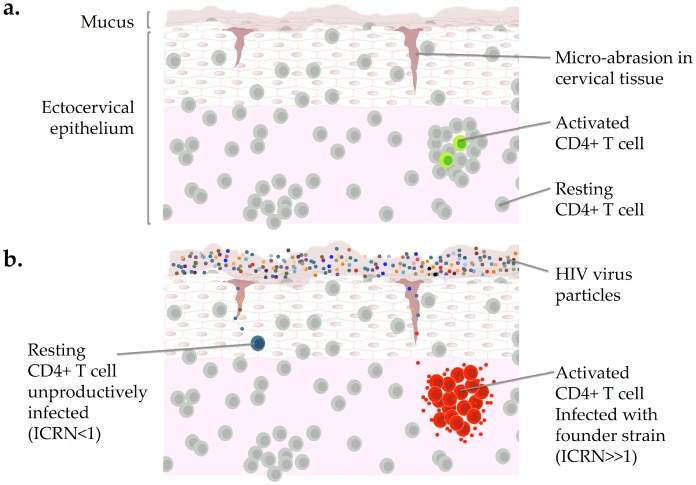
Heterogeneity among CD4+ T cells in the genital mucosa and the HIV founder strain. (A) The majority of CD4+ T cells in the female genital mucosa of an uninfected individual are resting cells; rare cells are activated. (B) Most virus particles remain trapped in the mucus that coats the cervical epithelium though a few can enter through microabrasions. Resting CD4+ T cells can become infected with HIV but do not produce infectious virus. Infection of activated CD4+ T cells, which tend to form clusters, have higher levels of gene expression than resting cells, and produce infectious virus, may be the superspreading event that establishes the HIV founder strain after sexual transmission. ICRN: individual cellular reproductive number. Based on data from references [3] and [10]. Images of cervical epithelium by OpenStax College [CC-BY-3.0 (http://creativecommons.org/licenses/by/3.0)] via Wikimedia Commons.

Experiments in a nonhuman primate model of HIV infection have demonstrated that the vast majority of CD4+ T cells in the genital mucosa of a healthy, uninfected individual are resting cells, which outnumber activated cells 70∶1 (Figure 2A) [9]. Activated CD4+ T cells express higher levels of CCR5 [33] and α4β7 [40] than do resting cells. In addition, experiments in nonhuman primates indicate that infected, activated cells contain five times more viral RNA, release 10-fold more viral particles to their surrounding environment, and tend to form larger cell clusters than resting cells [9]. Notably, although the nonhuman primate model has long been used to study many facets of HIV infection [53], the virus used in these experiments, SIV, expresses a protein that allows it to productively infect resting CD4+ T cells. In contrast, HIV-1 does not express this protein and is thus unable to generate productive infection in resting CD4+ T cells [54]. Resting human CD4+ T cells in the human genital mucosa are therefore even less likely to be able to produce and spread HIV than are resting CD4+ T cells in a nonhuman primate model.

Together, these data suggest that upon infection with HIV the majority of CD4+ T cells in the genital mucosa would spread the virus to few if any others and that only rare cells would have the capacity to release large quantities of HIV to their nearest neighbors. These rare cells may be activated CD4+ T cells, which have been described as “amplifiers” that can cause additional cells to become infected with HIV [10], [55]. Such rare cells may exhibit a specific set of traits that facilitate establishment of HIV inside the body. For example, experiments done in vitro indicate that CD4+ Th17 cells, which express high levels of CCR5 as well as the chemokine receptor CCR6 and the integrin α4β7, are preferentially targeted during early infection, and analysis of samples from female sex workers who are infected with HIV indicate that CD4+ Th17 cells are selectively depleted from the cervix during HIV infection [25], [56]–[58]. In addition, given that the majority of HIV infections begin with a single founding strain [59] and most infected cells in peripheral blood and lymph node tissue contain a single copy of HIV DNA (although these may or may not be representative of HIV integration in mucosal lymphatic cells) [51], infection of a single cell has the potential to establish HIV infection inside the genital mucosa after a given exposure. Infection of a rare CD4+ T cell with very high ICRN could thus be the superspreading event that both establishes HIV infection in the genital mucosa and selects a single founder strain (Figure 2B).

Such a founding superspreader infection event would parallel, for example, the dynamics that governed the SARS outbreak in Singapore, giving rise to an epidemic despite a low individual reproductive number for most individuals. As shown in Figure 1, infection of a single superspreading individual (labeled as “1”) triggered the SARS outbreak in Singapore in 2003.

## Implications of Cellular Superspreaders in HIV Infection

A highly skewed distribution of individual reproductive numbers in a population, in which most individuals infect few if any others but a tiny minority are superspreaders, has two important implications when applied to CD4+ T cells in the genital mucosa. First, since the majority of the cells would have a low ICRN, most, if not all, of the viral strains that successfully overcome physiological barriers during exposure are likely to infect cells that have an ICRN less than one. Provided that none of these cells becomes latently infected, which has been shown to occur within days after infection in vitro [60], [61] but has yet to be confirmed in vivo, a “local outbreak” inside the body would go extinct. In this case, infection of most cells immediately after sexual exposure would not lead to sustained infection, which could explain the very low infection-to-exposure ratio for sexual exposure to HIV.

Second, such short-lived “local outbreaks” of HIV within an individual could still yield a low level of virus production, even if the initial outbreak of HIV infection ultimately goes extinct. Among a group of nonhuman primates exposed to a low physiological dose of SIV, some animals experienced initial low levels of viral replication and immune response without ever proceeding to full infection or seroconversion, a phenomenon called occult infection [62]–[64]. In addition, some HIV-exposed, seronegative humans who continue to engage in high-risk sexual behavior exhibit immunological markers that indicate a prior immune response to HIV infection even though they remain seronegative, supporting the idea that local infections may have occurred in these patients [65]–[67]. Finally, analysis of the unsuccessful HIV vaccine STEP trial suggested that a large portion of the exposed individuals may have experienced occult infection, implying that this phenomenon could be more widespread than previously suspected [68]. There is, however, no direct experimental evidence for occult infection in humans.

If infection of a rare superspreader CD4+ T cell establishes the founder strain, then the few features of founder viral strains that have been observed might in fact confer a selective advantage during early infection. These features include shorter envelope glycoproteins with fewer N-linked glycosylation sites [69], [70] as well as preferential infection of CD4+ T cells expressing high levels of CCR5. Some founder viral strains have also exhibited high affinity for the α4β7 integrin receptor [71], [72] though this has not been universally observed [73], [5]. The founder virus, which more closely resembles the ancestral founder strain than it does the predominant strain in the transmitting partner [74], may be selected by its ability to infect a subtype of CD4+ T cell: a cellular superspreader. This specific efficiency could explain why HIV founder strains have not shown a consistent infectivity advantage in CD4+ T cells over strains from chronic infection in vitro [5], [75], [76], as these experiments likely do not fully replicate the cellular heterogeneity of the in vivo environment [32], [77], [78], where the founder viruses might have an advantage in infecting the rare superspreader cells.

## Conclusions

Here we have applied two key concepts from epidemiology: R_0_, which is the approximated product of number of contacts and shedding potential throughout the infectious period, and individual reproductive number, to suggest that a skewed distribution of individual cellular reproductive number among CD4+ T cells in the genital mucosa gives rise to cellular superspreaders that may drive establishment of HIV infection inside the genital mucosa after sexual transmission.

The definition of R_0_ provided here implicitly integrates transmission over the time that an index cell was infected, meaning that we have incorporated duration of infectiousness into our overall definition of R_0_. Thus, a CD4+ T cell could theoretically become a superspreader either by spreading a large amount of infectious virus to other cells in a short amount of time, or by spreading a smaller amount of virus to other cells for a comparatively longer period of time, or through some combination of the two. Though any of these mechanisms are possible in early infection [79], studies in nonhuman primates suggest that establishment of infection in the genital mucosa typically occurs within 3–7 days after male-to-female sexual exposure [62], [80]. The lifespan of a productively infected CD4+ T cell is on average 2.2 days [81]. Thus, in the specific case of HIV infection in the genital mucosal tissue, if infection becomes established via a superspreading event, it is likely to occur within the first few days of exposure and be driven by a relatively short-lived, productively infected cell that generates a much higher-than-average number of secondary infections due to a high shedding rate, or a high contact rate, or both. Notably, as in other superspreading events, establishment of HIV by infection of a cellular superspreader could occur even if the basic reproductive number for the entire population of susceptible cells is low.

If cellular HIV superspreaders do exist, and if they are the cellular culprits driving the establishment of HIV infection inside the body, then the most successful strategy for preventing the infection from becoming established in the body is to block or remove these cells before or shortly after infection in order to drive a local, within-host outbreak to extinction [13], [82]. Such cells may have specific traits, such as high expression of surface receptors including CCR5 and possibly also α4β7, that allow them to be identified and targeted by novel therapies to prevent establishment of infection. Several recent studies suggest that Th17 cells, a subset of CD4+ T cells, are preferentially infected by early viral strains and selectively depleted from the cervix during HIV infection [58], [25]. We are not aware of in vivo data that explicitly support the existence of cellular superspreaders; nevertheless, the data reviewed here suggest their existence, warranting further empirical research. Identification and targeting of cells most likely to become superspreaders could facilitate the development of preexposure or immediate postexposure therapies that could prevent a local outbreak of HIV inside the genital mucosa from becoming a within-host epidemic that spreads throughout the body [9], [83].

In this review, we have applied epidemiological concepts of disease spread specifically to explore unsolved questions regarding establishment of the HIV founder strain and the low infection-to-exposure ratio of infection after sexual transmission. We suggest that these concepts may also be applied more broadly to explain the documented existence of HIV founder strains after transmission via injection drug use and from mother to child [59] since CD4+ T cells in the blood of healthy, uninfected individuals are also heterogeneous, with only a very small subset exhibiting an activated or replicating phenotype [84].

Since heterogeneity among cells has been acknowledged as an important factor in a variety of cellular processes, including certain viral infections [12], we suggest that the epidemiological framework described here may also be applicable to the establishment and spread of other cellular diseases inside the body, including not only infections but perhaps also certain cancers. The impact of cellular heterogeneity may be particularly profound if the distribution of individual cellular reproductive numbers is highly skewed, yielding cellular superspreaders.

## References

[1] UNAIDS (2013) Global Report: UNAIDS report on the global AIDS epidemic 2013. 2013 edition. Online: Joint United Nations Programme on HIV/AIDS. Available: http://www.unaids.org/en/resources/campaigns/globalreport2013/globalreport/. Accessed 7 April 2014.

[2] Boily MC, Baggaley RF, Wang L, Masse B, White RG, et al (2009) Heterosexual risk of HIV-1 infection per sexual act: systematic review and meta-analysis of observational studies. Lancet Infect Dis 9: 118–129. 19179227 10.1016/S1473-3099(09)70021-0 PMC4467783

[3] Keele BF, Estes JD (2011) Barriers to mucosal transmission of immunodeficiency viruses. Blood 118: 839–846. 21555745 10.1182/blood-2010-12-325860 PMC3148165

[4] Li H, Bar KJ, Wang S, Decker JM, Chen Y, et al (2010) High Multiplicity Infection by HIV-1 in Men Who Have Sex with Men. PLoS Pathog 6: e1000890. 20485520 10.1371/journal.ppat.1000890 PMC2869329

[5] Parrish NF, Gao F, Li H, Giorgi EE, Barbian HJ, et al (2013) Phenotypic properties of transmitted founder HIV-1. Proc Natl Acad Sci U S A 110: 6626–6633. 23542380 10.1073/pnas.1304288110 PMC3637789

[6] Raj A, van Oudenaarden A (2008) Nature, nurture, or chance: stochastic gene expression and its consequences. Cell 135: 216–226. 18957198 10.1016/j.cell.2008.09.050 PMC3118044

[7] Sallusto F, Lanzavecchia A (2009) Heterogeneity of CD4+ memory T cells: functional modules for tailored immunity. Eur J Immunol 39: 2076–2082. 19672903 10.1002/eji.200939722

[8] Reilly C, Schacker T, Haase AT, Wietgrefe S, Krason D (2002) The Clustering of Infected SIV Cells in Lymphatic Tissue. J Am Stat Assoc 97: 943–954.

[9] Li Q, Estes JD, Schlievert PM, Duan L, Brosnahan AJ, et al (2009) Glycerol monolaurate prevents mucosal SIV transmission. Nature 458: 1034–1038. 19262509 10.1038/nature07831 PMC2785041

[10] Zhang ZQ, Wietgrefe SW, Li Q, Shore MD, Duan L, et al (2004) Roles of substrate availability and infection of resting and activated CD4+ T cells in transmission and acute simian immunodeficiency virus infection. Proc Natl Acad Sci U S A 101: 5640–5645. 15064398 10.1073/pnas.0308425101 PMC397458

[11] Zhu J, Paul WE (2010) Heterogeneity and plasticity of T helper cells. Cell Res 20: 4–12. 20010916 10.1038/cr.2009.138 PMC3494736

[12] Altschuler SJ, Wu LF (2010) Cellular heterogeneity: do differences make a difference? Cell 141: 559–563. 20478246 10.1016/j.cell.2010.04.033 PMC2918286

[13] McKinnon LR, Kaul R (2012) Quality and quantity: mucosal CD4+ T cells and HIV susceptibility. Curr Opin HIV AIDS 7: 195–202. 22314505 10.1097/COH.0b013e3283504941

[14] Lloyd-Smith JO, Schreiber SJ, Kopp PE, Getz WM (2005) Superspreading and the effect of individual variation on disease emergence. Nature 438: 355–359. 16292310 10.1038/nature04153 PMC7094981

[15] Anderson RM, May RM (1991) Infectious diseases of humans: dynamics and control. New York: Oxford University Press.

[16] Keeling MJ, Rohani P (2008) Modeling infectious diseases in humans and animals. Princeton: Princeton University Press. xi, 366 pp.

[17] De Serres G, Markowski F, Toth E, Landry M, Auger D, et al (2013) Largest measles epidemic in North America in a decade–Quebec, Canada, 2011: contribution of susceptibility, serendipity, and superspreading events. J Infect Dis 207: 990–998. 23264672 10.1093/infdis/jis923

[18] Stein RA (2011) Super-spreaders in infectious diseases. Int J Infect Dis 15: e510–513. 21737332 10.1016/j.ijid.2010.06.020 PMC7110524

[19] Centers for Disease Control and Prevention (2003) Severe acute respiratory syndrome–Singapore, 2003. MMWR Morb Mortal Wkly Rep 52: 405–411. 12807088

[20] Ribeiro RM, Qin L, Chavez LL, Li D, Self SG, et al (2010) Estimation of the initial viral growth rate and basic reproductive number during acute HIV-1 infection. J Virol 84: 6096–6102. 20357090 10.1128/JVI.00127-10 PMC2876646

[21] Anderson D, Politch JA, Pudney J (2011) HIV infection and immune defense of the penis. Am J Reprod Immunol 65: 220–229. 21214659 10.1111/j.1600-0897.2010.00941.x PMC3076079

[22] Liu A, Yang Y, Liu L, Meng Z, Li L, et al (2014) Differential Compartmentalization of HIV-Targeting Immune Cells in Inner and Outer Foreskin Tissue. PLoS One 9: e85176. 24454812 10.1371/journal.pone.0085176 PMC3893184

[23] Patterson BK, Landay A, Siegel JN, Flener Z, Pessis D, et al (2002) Susceptibility to human immunodeficiency virus-1 infection of human foreskin and cervical tissue grown in explant culture. Am J Pathol 161: 867–873. 12213715 10.1016/S0002-9440(10)64247-2 PMC1867269

[24] Saba E, Grivel JC, Vanpouille C, Brichacek B, Fitzgerald W, et al (2010) HIV-1 sexual transmission: early events of HIV-1 infection of human cervico-vaginal tissue in an optimized ex vivo model. Mucosal Immunol 3: 280–290. 20147895 10.1038/mi.2010.2 PMC3173980

[25] McKinnon LR, Nyanga B, Chege D, Izulla P, Kimani M, et al (2011) Characterization of a human cervical CD4+ T cell subset coexpressing multiple markers of HIV susceptibility. J Immunol 187: 6032–6042. 22048765 10.4049/jimmunol.1101836

[26] Grivel JC, Elliott J, Lisco A, Biancotto A, Condack C, et al (2007) HIV-1 pathogenesis differs in rectosigmoid and tonsillar tissues infected ex vivo with CCR5- and CXCR4-tropic HIV-1. AIDS 21: 1263–1272. 17545702 10.1097/QAD.0b013e3281864667

[27] Pudney J, Quayle AJ, Anderson DJ (2005) Immunological microenvironments in the human vagina and cervix: mediators of cellular immunity are concentrated in the cervical transformation zone. Biol Reprod 73: 1253–1263. 16093359 10.1095/biolreprod.105.043133

[28] Strain MC, Richman DD, Wong JK, Levine H (2002) Spatiotemporal dynamics of HIV propagation. J Theor Biol 218: 85–96. 12297072 10.1006/jtbi.2002.3055

[29] Snijder B, Sacher R, Ramo P, Damm EM, Liberali P, et al (2009) Population context determines cell-to-cell variability in endocytosis and virus infection. Nature 461: 520–523. 19710653 10.1038/nature08282

[30] Snijder B, Sacher R, Ramo P, Liberali P, Mench K, et al (2012) Single-cell analysis of population context advances RNAi screening at multiple levels. Mol Syst Biol 8: 579. 22531119 10.1038/msb.2012.9 PMC3361004

[31] Zhong P, Agosto LM, Munro JB, Mothes W (2013) Cell-to-cell transmission of viruses. Curr Opin Virol 3: 44–50. 23219376 10.1016/j.coviro.2012.11.004 PMC3587356

[32] Sagar M (2010) HIV-1 transmission biology: selection and characteristics of infecting viruses. J Infect Dis 202 Suppl 2: S289–296. 20846035 10.1086/655656 PMC2946383

[33] Lee B, Sharron M, Montaner LJ, Weissman D, Doms RW (1999) Quantification of CD4, CCR5, and CXCR4 levels on lymphocyte subsets, dendritic cells, and differentially conditioned monocyte-derived macrophages. Proc Natl Acad Sci U S A 96: 5215–5220. 10220446 10.1073/pnas.96.9.5215 PMC21844

[34] Chen J, Dang Q, Unutmaz D, Pathak VK, Maldarelli F, et al (2005) Mechanisms of nonrandom human immunodeficiency virus type 1 infection and double infection: preference in virus entry is important but is not the sole factor. J Virol 79: 4140–4149. 15767415 10.1128/JVI.79.7.4140-4149.2005 PMC1061529

[35] Kabat D, Kozak SL, Wehrly K, Chesebro B (1994) Differences in CD4 dependence for infectivity of laboratory-adapted and primary patient isolates of human immunodeficiency virus type 1. J Virol 68: 2570–2577. 8139036 10.1128/jvi.68.4.2570-2577.1994 PMC236734

[36] Parker ZF, Iyer SS, Wilen CB, Parrish NF, Chikere KC, et al (2013) Transmitted/founder and chronic HIV-1 envelope proteins are distinguished by differential utilization of CCR5. J Virol 87: 2401–2411. 23269796 10.1128/JVI.02964-12 PMC3571396

[37] Johnston SH, Lobritz MA, Nguyen S, Lassen K, Delair S, et al (2009) A quantitative affinity-profiling system that reveals distinct CD4/CCR5 usage patterns among human immunodeficiency virus type 1 and simian immunodeficiency virus strains. J Virol 83: 11016–11026. 19692480 10.1128/JVI.01242-09 PMC2772777

[38] Bozek K, Eckhardt M, Sierra S, Anders M, Kaiser R, et al (2012) An expanded model of HIV cell entry phenotype based on multi-parameter single-cell data. Retrovirology 9: 60. 22830600 10.1186/1742-4690-9-60 PMC3464718

[39] Arthos J, Cicala C, Martinelli E, Macleod K, Van Ryk D, et al (2008) HIV-1 envelope protein binds to and signals through integrin alpha4beta7, the gut mucosal homing receptor for peripheral T cells. Nat Immunol 9: 301–309. 18264102 10.1038/ni1566

[40] Cicala C, Martinelli E, McNally JP, Goode DJ, Gopaul R, et al (2009) The integrin alpha4beta7 forms a complex with cell-surface CD4 and defines a T-cell subset that is highly susceptible to infection by HIV-1. Proc Natl Acad Sci U S A 106: 20877–20882. 19933330 10.1073/pnas.0911796106 PMC2780317

[41] Harris RS, Hultquist JF, Evans DT (2012) The restriction factors of human immunodeficiency virus. J Biol Chem 287: 40875–40883. 23043100 10.1074/jbc.R112.416925 PMC3510791

[42] Wang X, Abudu A, Son S, Dang Y, Venta PJ, et al (2011) Analysis of human APOBEC3H haplotypes and anti-human immunodeficiency virus type 1 activity. J Virol 85: 3142–3152. 21270145 10.1128/JVI.02049-10 PMC3067873

[43] Mous K, Jennes W, De Roo A, Pintelon I, Kestens L, et al (2011) Intracellular detection of differential APOBEC3G, TRIM5alpha, and LEDGF/p75 protein expression in peripheral blood by flow cytometry. J Immunol Methods 372: 52–64. 21784078 10.1016/j.jim.2011.06.028

[44] Pan X, Baldauf HM, Keppler OT, Fackler OT (2013) Restrictions to HIV-1 replication in resting CD4(+) T lymphocytes. Cell Res 23: 876–885. 23732522 10.1038/cr.2013.74 PMC3698640

[45] Marquardt A, Halle S, Seckert CK, Lemmermann NA, Veres TZ, et al (2011) Single cell detection of latent cytomegalovirus reactivation in host tissue. J Gen Virol 92: 1279–1291. 21325477 10.1099/vir.0.029827-0

[46] Weinberger LS, Burnett JC, Toettcher JE, Arkin AP, Schaffer DV (2005) Stochastic gene expression in a lentiviral positive-feedback loop: HIV-1 Tat fluctuations drive phenotypic diversity. Cell 122: 169–182. 16051143 10.1016/j.cell.2005.06.006

[47] Schroder AR, Shinn P, Chen H, Berry C, Ecker JR, et al (2002) HIV-1 integration in the human genome favors active genes and local hotspots. Cell 110: 521–529. 12202041 10.1016/s0092-8674(02)00864-4

[48] Mitchell RS, Beitzel BF, Schroder AR, Shinn P, Chen H, et al (2004) Retroviral DNA integration: ASLV, HIV, and MLV show distinct target site preferences. PLoS Biol 2: e234. 15314653 10.1371/journal.pbio.0020234 PMC509299

[49] Wu X, Li Y, Crise B, Burgess SM (2003) Transcription start regions in the human genome are favored targets for MLV integration. Science 300: 1749–1751. 12805549 10.1126/science.1083413

[50] Skupsky R, Burnett JC, Foley JE, Schaffer DV, Arkin AP (2010) HIV promoter integration site primarily modulates transcriptional burst size rather than frequency. PLoS Comput Biol 6: e1000952. 20941390 10.1371/journal.pcbi.1000952 PMC2947985

[51] Josefsson L, Palmer S, Faria NR, Lemey P, Casazza J, et al (2013) Single cell analysis of lymph node tissue from HIV-1 infected patients reveals that the majority of CD4+ T-cells contain one HIV-1 DNA molecule. PLoS Pathog 9: e1003432. 23818847 10.1371/journal.ppat.1003432 PMC3688524

[52] Jung A, Maier R, Vartanian JP, Bocharov G, Jung V, et al (2002) Recombination: Multiply infected spleen cells in HIV patients. Nature 418: 144. 12110879 10.1038/418144a

[53] Haigwood NL (2009) Update on animal models for HIV research. Eur J Immunol 39: 1994–1999. 19672890 10.1002/eji.200939576 PMC2866064

[54] Pan LZ, Werner A, Levy JA (1993) Detection of plasma viremia in human immunodeficiency virus-infected individuals at all clinical stages. J Clin Microbiol 31: 283–288. 8094395 10.1128/jcm.31.2.283-288.1993 PMC262750

[55] Miller CJ, Marthas M, Torten J, Alexander NJ, Moore JP, et al (1994) Intravaginal inoculation of rhesus macaques with cell-free simian immunodeficiency virus results in persistent or transient viremia. J Virol 68: 6391–6400. 8083977 10.1128/jvi.68.10.6391-6400.1994 PMC237059

[56] El Hed A, Khaitan A, Kozhaya L, Manel N, Daskalakis D, et al (2010) Susceptibility of human Th17 cells to human immunodeficiency virus and their perturbation during infection. J Infect Dis 201: 843–854. 20144043 10.1086/651021 PMC2849315

[57] Monteiro P, Gosselin A, Wacleche VS, El-Far M, Said EA, et al (2011) Memory CCR6+CD4+ T cells are preferential targets for productive HIV type 1 infection regardless of their expression of integrin beta7. J Immunol 186: 4618–4630. 21398606 10.4049/jimmunol.1004151

[58] Alvarez Y, Tuen M, Shen G, Nawaz F, Arthos J, et al (2013) Preferential HIV infection of CCR6+ Th17 cells is associated with higher levels of virus receptor expression and lack of CCR5 ligands. J Virol 87: 10843–10854. 23903844 10.1128/JVI.01838-13 PMC3807416

[59] Shaw GM, Hunter E (2012) HIV transmission. Cold Spring Harb Perspect Med 2: a006965. 23043157 10.1101/cshperspect.a006965 PMC3543106

[60] Dahabieh MS, Ooms M, Simon V, Sadowski I (2013) A double-fluorescent HIV-1 reporter shows that the majority of integrated HIV-1 is latent shortly after infection. J Virol 87: 4716–4727. 23408629 10.1128/JVI.03478-12 PMC3624398

[61] Duverger A, Jones J, May J, Bibollet-Ruche F, Wagner FA, et al (2009) Determinants of the establishment of human immunodeficiency virus type 1 latency. J Virol 83: 3078–3093. 19144703 10.1128/JVI.02058-08 PMC2655589

[62] Miller CJ, Li Q, Abel K, Kim EY, Ma ZM, et al (2005) Propagation and dissemination of infection after vaginal transmission of simian immunodeficiency virus. J Virol 79: 9217–9227. 15994816 10.1128/JVI.79.14.9217-9227.2005 PMC1168785

[63] McChesney MB, Collins JR, Lu D, Lu X, Torten J, et al (1998) Occult systemic infection and persistent simian immunodeficiency virus (SIV)-specific CD4(+)-T-cell proliferative responses in rhesus macaques that were transiently viremic after intravaginal inoculation of SIV. J Virol 72: 10029–10035. 9811741 10.1128/jvi.72.12.10029-10035.1998 PMC110525

[64] Ma ZM, Abel K, Rourke T, Wang Y, Miller CJ (2004) A period of transient viremia and occult infection precedes persistent viremia and antiviral immune responses during multiple low-dose intravaginal simian immunodeficiency virus inoculations. J Virol 78: 14048–14052. 15564513 10.1128/JVI.78.24.14048-14052.2004 PMC533914

[65] Beyrer C, Artenstein AW, Rugpao S, Stephens H, VanCott TC, et al (1999) Epidemiologic and biologic characterization of a cohort of human immunodeficiency virus type 1 highly exposed, persistently seronegative female sex workers in northern Thailand. Chiang Mai HEPS Working Group. J Infect Dis 179: 59–67. 9841823 10.1086/314556

[66] Kaul R, Rowland-Jones SL, Kimani J, Fowke K, Dong T, et al (2001) New insights into HIV-1 specific cytotoxic T-lymphocyte responses in exposed, persistently seronegative Kenyan sex workers. Immunol Lett 79: 3–13. 11595284 10.1016/s0165-2478(01)00260-7

[67] Horton RE, Ball TB, Wachichi C, Jaoko W, Rutherford WJ, et al (2009) Cervical HIV-specific IgA in a population of commercial sex workers correlates with repeated exposure but not resistance to HIV. AIDS Res Hum Retroviruses 25: 83–92. 19108692 10.1089/aid.2008.0207

[68] Uberla K (2008) HIV vaccine development in the aftermath of the STEP study: re-focus on occult HIV infection? PLoS Pathog 4: e1000114. 18769723 10.1371/journal.ppat.1000114 PMC2517652

[69] Go EP, Hewawasam G, Liao HX, Chen H, Ping LH, et al (2011) Characterization of glycosylation profiles of HIV-1 transmitted/founder envelopes by mass spectrometry. J Virol 85: 8270–8284. 21653661 10.1128/JVI.05053-11 PMC3147976

[70] Gnanakaran S, Bhattacharya T, Daniels M, Keele BF, Hraber PT, et al (2011) Recurrent signature patterns in HIV-1 B clade envelope glycoproteins associated with either early or chronic infections. PLoS Pathog 7: e1002209. 21980282 10.1371/journal.ppat.1002209 PMC3182927

[71] Nawaz F, Cicala C, Van Ryk D, Block KE, Jelicic K, et al (2011) The genotype of early-transmitting HIV gp120s promotes alpha (4) beta(7)-reactivity, revealing alpha (4) beta(7) +/CD4+ T cells as key targets in mucosal transmission. PLoS Pathog 7: e1001301. 21383973 10.1371/journal.ppat.1001301 PMC3044691

[72] Pena-Cruz V, Etemad B, Chatziandreou N, Nyein PH, Stock S, et al (2013) HIV-1 envelope replication and alpha4beta7 utilization among newly infected subjects and their corresponding heterosexual partners. Retrovirology 10: 162. 24369910 10.1186/1742-4690-10-162 PMC3883469

[73] Etemad B, Gonzalez OA, McDonough S, Pena-Cruz V, Sagar M (2013) Early infection HIV-1 envelope V1-V2 genotypes do not enhance binding or replication in cells expressing high levels of alpha4beta7 integrin. J Acquir Immune Defic Syndr 64: 249–253. 23797693 10.1097/QAI.0b013e3182a06ddd PMC3800220

[74] Redd AD, Collinson-Streng AN, Chatziandreou N, Mullis CE, Laeyendecker O, et al (2012) Previously transmitted HIV-1 strains are preferentially selected during subsequent sexual transmissions. J Infect Dis 206: 1433–1442. 22997233 10.1093/infdis/jis503 PMC3466994

[75] Parrish NF, Wilen CB, Banks LB, Iyer SS, Pfaff JM, et al (2012) Transmitted/founder and chronic subtype C HIV-1 use CD4 and CCR5 receptors with equal efficiency and are not inhibited by blocking the integrin alpha4beta7. PLoS Pathog 8: e1002686. 22693444 10.1371/journal.ppat.1002686 PMC3364951

[76] Mota TM, Murray JM, Center RJ, Purcell DF, McCaw JM (2012) Application of a case-control study design to investigate genotypic signatures of HIV-1 transmission. Retrovirology 9: 54. 22731404 10.1186/1742-4690-9-54 PMC3419081

[77] Shen C, Ding M, Ratner D, Montelaro RC, Chen Y, et al (2012) Evaluation of cervical mucosa in transmission bottleneck during acute HIV-1 infection using a cervical tissue-based organ culture. PLoS One 7: e32539. 22412886 10.1371/journal.pone.0032539 PMC3296723

[78] Ochsenbauer C, Edmonds TG, Ding H, Keele BF, Decker J, et al (2012) Generation of transmitted/founder HIV-1 infectious molecular clones and characterization of their replication capacity in CD4 T lymphocytes and monocyte-derived macrophages. J Virol 86: 2715–2728. 22190722 10.1128/JVI.06157-11 PMC3302286

[79] Pearson JE, Krapivsky P, Perelson AS (2011) Stochastic theory of early viral infection: continuous versus burst production of virions. PLoS Comput Biol 7: e1001058. 21304934 10.1371/journal.pcbi.1001058 PMC3033366

[80] Zhang Z, Schuler T, Zupancic M, Wietgrefe S, Staskus KA, et al (1999) Sexual transmission and propagation of SIV and HIV in resting and activated CD4+ T cells. Science 286: 1353–1357. 10558989 10.1126/science.286.5443.1353

[81] Perelson AS, Neumann AU, Markowitz M, Leonard JM, Ho DD (1996) HIV-1 dynamics in vivo: virion clearance rate, infected cell life-span, and viral generation time. Science 271: 1582–1586. 8599114 10.1126/science.271.5255.1582

[82] Archin NM, Vaidya NK, Kuruc JD, Liberty AL, Wiegand A, et al (2012) Immediate antiviral therapy appears to restrict resting CD4+ cell HIV-1 infection without accelerating the decay of latent infection. Proc Natl Acad Sci U S A 109: 9523–9528. 22645358 10.1073/pnas.1120248109 PMC3386138

[83] Haase AT (2011) Early events in sexual transmission of HIV and SIV and opportunities for interventions. Annu Rev Med 62: 127–139. 21054171 10.1146/annurev-med-080709-124959

[84] Funderburg NT, Stubblefield Park SR, Sung HC, Hardy G, Clagett B, et al (2013) Circulating CD4(+) and CD8(+) T cells are activated in inflammatory bowel disease and are associated with plasma markers of inflammation. Immunology 140: 87–97. 23600521 10.1111/imm.12114 PMC3809709

[85] Chowell G, Hengartner NW, Castillo-Chavez C, Fenimore PW, Hyman JM (2004) The basic reproductive number of Ebola and the effects of public health measures: the cases of Congo and Uganda. J Theor Biol 229: 119–126. 15178190 10.1016/j.jtbi.2004.03.006

[86] Khan AS, Tshioko FK, Heymann DL, Le Guenno B, Nabeth P, et al (1999) The reemergence of Ebola hemorrhagic fever, Democratic Republic of the Congo, 1995. Commission de Lutte contre les Epidemies a Kikwit. J Infect Dis 179 Suppl 1: S76–86. 9988168 10.1086/514306

[87] Christensen PE, Schmidt H, Bang HO, Andersen V, Jordal B, et al (1953) An epidemic of measles in southern Greenland, 1951; measles in virgin soil. II. The epidemic proper. Acta Med Scand 144: 430–449. 13050347 10.1111/j.0954-6820.1953.tb15717.x

[88] Chen RT, Goldbaum GM, Wassilak SG, Markowitz LE, Orenstein WA (1989) An explosive point-source measles outbreak in a highly vaccinated population. Modes of transmission and risk factors for disease. Am J Epidemiol 129: 173–182. 2910058 10.1093/oxfordjournals.aje.a115106

[89] Gani R, Leach S (2004) Epidemiologic determinants for modeling pneumonic plague outbreaks. Emerg Infect Dis 10: 608–614. 15200849 10.3201/eid1004.030509 PMC3323083

[90] Tieh TH, Landauer E, et al (1948) Primary pneumonic plague in Mukden, 1946, and report of 39 cases with three recoveries. J Infect Dis 82: 52–58. 18898004 10.1093/infdis/82.1.52

[91] Bauch CT, Lloyd-Smith JO, Coffee MP, Galvani AP (2005) Dynamically modeling SARS and other newly emerging respiratory illnesses: past, present, and future. Epidemiology 16: 791–801. 16222170 10.1097/01.ede.0000181633.80269.4c

[92] Yu IT, Li Y, Wong TW, Tam W, Chan AT, et al (2004) Evidence of airborne transmission of the severe acute respiratory syndrome virus. N Engl J Med 350: 1731–1739. 15102999 10.1056/NEJMoa032867

[93] Kamps BS, Hoffmann C, editors (2005) SARS Reference. 3rd edition. Flying Publisher.

[94] Varia M, Wilson S, Sarwal S, McGeer A, Gournis E, et al (2003) Investigation of a nosocomial outbreak of severe acute respiratory syndrome (SARS) in Toronto, Canada. CMAJ 169: 285–292. 12925421 PMC180651

[95] Gani R, Leach S (2001) Transmission potential of smallpox in contemporary populations. Nature 414: 748–751. 11742399 10.1038/414748a

[96] Fenner F (1988) Smallpox and its eradication. Geneva: World Health Organization. xvi, 1460 pp.

